# TEMPORAL TRENDS IN INCIDENCE RATE OF DIFFERENT TYPES OF MULTIMORBIDITY AMONG MIDDLE- AND OLD-AGED AMERICANS, 2000–2020

**DOI:** 10.1093/geroni/igad104.0132

**Published:** 2023-12-21

## Abstract

This study aimed to examine secular trends in multimorbidity incidence rates among middle and old aged American adults. Participants aged ≥ 50 years from the Health and Retirement Study (HRS) during 2000-2020 were divided into nine consecutive and overlapping cohorts. Four types of multimorbidity (general multimorbidity, physical multimorbidity, multimorbidity including psychiatric disorders, and cardiometabolic multimorbidity) were identified based on seven self-reported physical diseases and two psychiatric conditions confirmed by validated scales. Generalized estimating equation (GEE) models incorporating a restricted cubic spline were used to examine adjusted nonlinear trends. Joinpoint regression models were adapted to estimate annual percentage changes and determine the contributions of baseline characteristics to the trends. The adjusted general multimorbidity incidence rate (per 1,000 person-years) increased from 76.0 (95% confidence interval(CI) = 71.8-80.4) in 2000-2004 to 112.2 (95% CI = 106.4-118.4) in 2016-2020. Both general multimorbidity and multimorbidity including psychiatric disorders showed a rising trend followed by a period of slight decrease from 2006-2010 to 2010-2014 and then a sustained increase, while physical multimorbidity and cardiometabolic multimorbidity showed a similar trend of increasing till 2006-2010 and then a relative plateau. Race/ethnicity, socioeconomic status, marital status, and dementia were significant contributors to changes in the incidence rate during 2000-2020. The incidence rate of each type of multimorbidity showed different fluctuations among middle and old aged adults in the US from 2000 to 2020. Findings inform clinicians, policymakers, and researchers to develop targeted interventions and provide comprehensive multimorbidity management in community settings.

